# Clinical and economic burden of respiratory syncytial virus in Spanish children: the BARI study

**DOI:** 10.1186/s12879-022-07745-0

**Published:** 2022-09-29

**Authors:** F. Martinón-Torres, M. Carmo, L. Platero, G. Drago, J. L. López-Belmonte, M. Bangert, J. Díez-Domingo, M. Garcés-Sánchez

**Affiliations:** 1 Translational Pediatrics and Infectious Diseases, Hospital Clínico Universitario and Universidad de Santiago de Compostela, Galicia, Spain; 2 Genetics, Vaccines and Pediatric Infectious Diseases Research Group (GENVIP), Instituto de Investigación Sanitaria de Santiago and Universidad de Santiago de Compostela (USC), Rúa da Choupana, s/n, Santiago de Compostela, Galicia, 15706 A Coruña, Spain; 3 CIBER Enfermedades Respiratorias (CIBERES), Instituto de Salud Carlos III, Madrid, Spain; 4 IQVIA, Barcelona, Spain; 5 Sanofi, Madrid, Spain; 6 Sanofi, Barcelona, Spain; 7 Sanofi, Lyon, France; 8 Vaccine Research Department, FISABIO, Valencia, Spain; 9 Pediatrics Service, Nazaret Health Center, Valencia, Spain

**Keywords:** Respiratory syncytial virus, Bronchiolitis, Burden, Epidemiology, Children, Acute lower respiratory infection

## Abstract

Respiratory syncytial virus (RSV) infection is a major cause of morbidity in children. However, its disease burden remains poorly understood, particularly outside of the hospital setting. Our study aimed to estimate the burden of medically attended acute lower respiratory infection (ALRI) cases potentially related to RSV in Spanish children. Longitudinal data from September 2017 to June 2018 of 51,292 children aged < 5 years old from the National Healthcare System (NHS) of two Spanish regions were used. Three case definitions were considered: (a) RSV-specific; (b) RSV-specific and unspecified acute bronchiolitis (RSV-specific and Bronchiolitis), and; (c) RSV-specific and unspecified ALRI (RSV-specific and ALRI). A total of 3460 medically attended ALRI cases potentially due to RSV were identified, of which 257 (7.4%), 164 (4.7%), and 3039 (87.8%) coded with RSV-specific, unspecific bronchiolitis, and unspecific ALRI codes, respectively. Medically attended RSV-specific and ALRI cases per 1000 children was 134.4 in the first year of life, 119.4 in the second, and 35.3 between 2 and 5 years old. Most cases were observed in otherwise healthy children (93.1%). Mean direct healthcare cost per medically attended RSV-specific and ALRI case was €1753 in the first year of life, €896 in the second, and €683 between 2 and 5 years old. Hospitalization was the main driver of these costs, accounting for 55.6%, 38.0% and 33.4%, in each respective age group. In RSV-specific cases, mean direct healthcare cost per medically attended case was higher, mostly due to hospitalization: €3362 in the first year of life (72.9% from hospitalizations), €3252 in the second (72.1%), and €3514 between 2 and 5 years old (74.2%). These findings suggest that hospitalization data alone will underestimate the RSV infections requiring medical care, as will relying only on RSV-specific codes. RSV testing and codification must be improved and preventive solutions adopted, to protect all infants, particularly during the first year of life.

**Supplementary Information:**

The online version contains supplementary material available at 10.1186/s12879-022-07745-0.

## Background

Respiratory syncytial virus (RSV) infection is a global public health challenge and a major cause of mortality and morbidity in children, specially infants [1–3]. Clinical manifestations associated with RSV infection that usually drive demand of healthcare resources in children are bronchiolitis, pneumonia and other acute lower respiratory infections [4]. Young age and being born preterm or having medical conditions such as congenital heart disease, bronchopulmonary dysplasia, malformations, neuromuscular diseases, or immunological disorders are known risk factors for severe complications [4, 5], although most cases are observed in otherwise healthy children born at term [6–8]. The RSV Evidence-a Geographical Archive of the Literature (REGAL) series estimated that, in the Western countries, RSV was associated with 12–63% of all acute respiratory infections (ARIs) and 19–81% of all viral ARIs leading to hospitalizations in children [1].

The near advent of RSV preventive tools has increased the priority on understanding the real-world burden caused by this infectious agent, in order to support the assessment of new preventive options, once they become available [9]. However, several knowledge gaps still exist and substantial variation is found in reported incidence rates across studies [4]. On one hand, studies often include only RSV-specific and/or acute bronchiolitis diagnosis codes—or RSV confirmed cases when laboratory data is available—approaches which are expected to widely underestimate the number of RSV cases, due to lack of systematic testing and coding [10–12]. On the other hand, most studies focus on hospitalizations, leaving outside substantial direct healthcare burden from outpatient visits [7, 13–15]. In United States, a study found the incidence of RSV cases in the outpatient setting to be over thirty times higher than the incidence of hospitalizations [7]. In Spain, Quiles et al. reported that, in Valencia, nearly 90% of the bronchiolitis cases in children < 2 years were managed in outpatient settings [13].

The objective of this study was to estimate the incidence and burden (clinical and economic) of medically attended acute lower respiratory infection (ALRI) cases potentially related to RSV in Spanish children aged < 5 years old in two Spanish regions and assessing the impact of using distinct RSV definitions.

## Methods

### Study design

The Burden of Acute Respiratory Infections (BARI) study is a multidimensional real-world evidence study assessing the clinical and economic burden of acute respiratory infections (influenza and respiratory syncytial virus) in Spain. We are reporting here the results for a retrospective cost-of-illness analysis conducted using data from a longitudinal electronic medical records database from two Spanish regions to estimate the direct healthcare cost per medically attended potential RSV case in children aged < 5 years old during the epidemic season 2017/18, from the perspective of the Spanish National Health Service (NHS).

### Database

This study used an IQVIA database including anonymized data extracted from the electronic medical records (EMR) of four Spanish regions, available between January 2017 and December 2018. Due to confidentiality agreements in place, the specific regions included in the database cannot be disclosed. The information collected in the database is provided by the regions themselves.

**Fig. 1:**
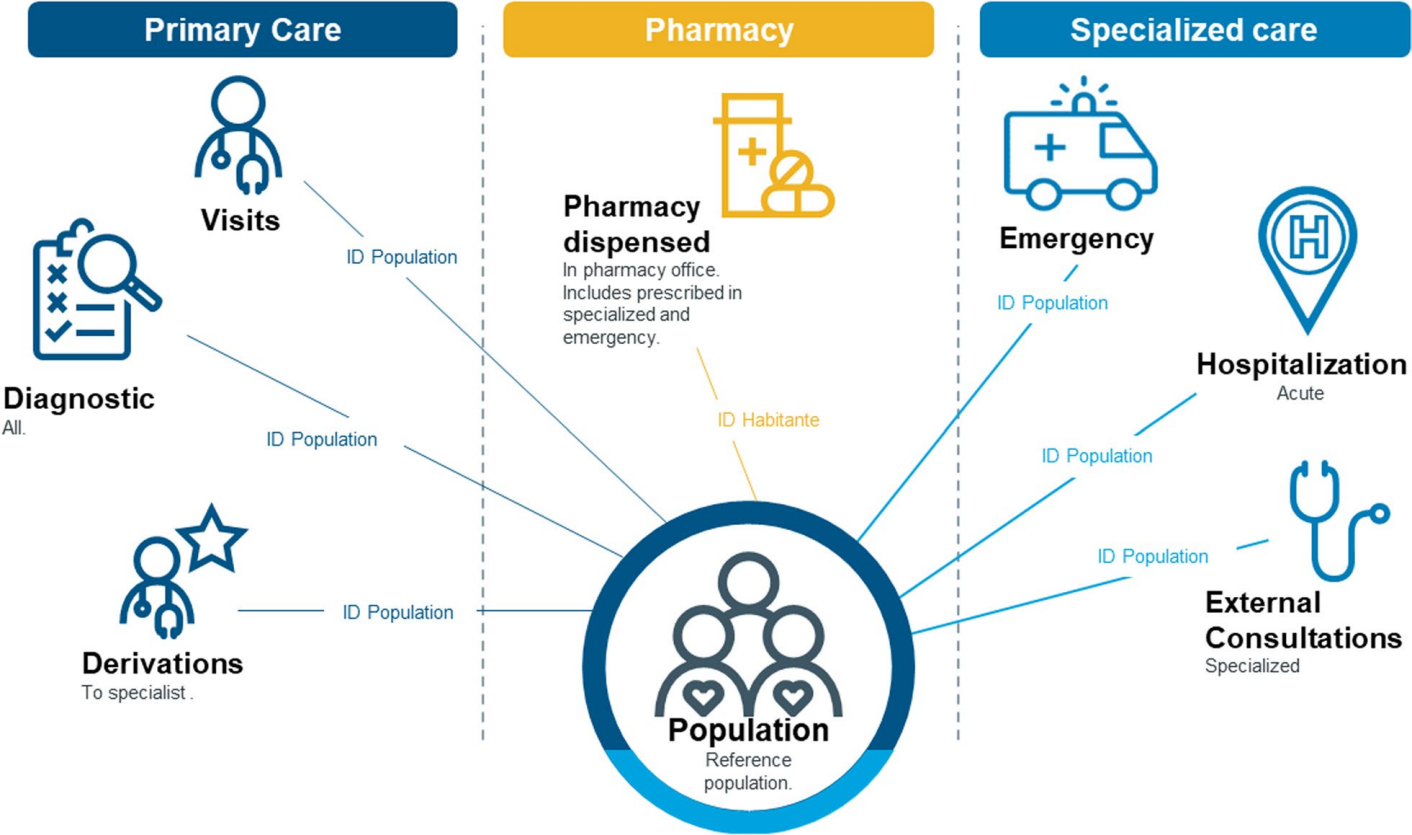
Details of the database used in the study

This database includes patients’ characteristics (age, gender), all their visits to distinct NHS healthcare providers and their diagnosis leading to the healthcare visit—using the International Classification of Diseases 9th Revision (ICD-9-MC) and 10th Revision (ICD-10-ES) and International Classification for Primary Care (ICPC-2), depending on the region—as well as related comorbidities or other significant diseases. It enables a traceability of resource consumption per patient across distinct healthcare settings, namely including information from primary care general practitioners and nurses activities, specialized care (outpatient’s consultations), visits to the emergency department, hospitalizations and retail pharmaceutical products prescribed by physicians. Information on acute and chronic diagnoses with date of diagnostic is available for every inhabitant, thus enabling the identification of individuals who had a potential RSV diagnosis as well as other medical conditions (risk factors). All the contacts that an inhabitant has had throughout the period are assigned to the inhabitant, so that the intensity of the contacts at the different care levels can be identified (Fig. 1).

As at 31st December 2018, the database contained longitudinal data of 1.9 million inhabitants from four Spanish areas, of which 82,652 (4.4%) were aged < 5 years old. Only the two regions who use only ICD codes across all settings of care were included in this study, as ICPC-2 codes did not enable our three RSV case definitions to be used. This resulted in data from 51,292 children aged < 5 years old being included in this study.

### RSV case definition

Study population included child having an RSV-related event based on ICD-9-CM and ICD-10-ES codes used in the diagnosis, regardless if classified as a primary or secondary diagnosis, according to three distinct case definitions (Table 1): (a) RSV-specific; (b) RSV-specific and unspecified acute bronchiolitis (RSV-specific and Bronchiolitis), and; (c) RSV-specific and unspecified ALRI (RSV-specific and ALRI). Unless otherwise stated, results were presented for the broadest definition (RSV-specific and ALRI). These case definitions were based on findings from Cai et al., who reported a higher sensitivity for these broader definitions than RSV-specific ICD-10 codes, without sacrificing specificity [12].

**Table 1:** List of ICD-9 and ICD-10 diagnostic codes used to identify respiratory syncytial virus cases

			Description	ICD-9	ICD-10
RSV-specific and ALRI	RSV-specific and bronchiolitis	RSV-specific	RSV	079.6	N/A
			Acute bronchiolitis due to RSV	466.11	J21.0
			Pneumonia due to RSV	480.1	J12.1
			RSV as the cause of diseases classified elsewhere	N/A	B97.4
			Acute bronchitis due to RSV	N/A	J20.5
			Acute bronchiolitis due to other infectious organisms	466.19	J21.8
			Acute bronchiolitis, unspecified		J21.9
			Acute bronchitis due to other infectious organisms	466.0	J20.8
			Acute bronchitis, unspecified		J20.9
			Unspecified ALRI	519.8	J22
			Viral pneumonia, not elsewhere classified	480.3;480.8; 480.9; 487.0	J12.8; J12.9
			Pneumonia, organism unspecified	485; 486	J18.0, J18.8, J18.9

*ALRI *acute lower respiratory infection, *N/A* not applicable, *RSV* respiratory syncytial virus

Population was stratified by age group [0–1] month, [1–2] months, [2–3] months, [3–6] months, [6–12] months, [12–24] months, [24–36] months and [36–60] months; [0–24] and [24–60] months; and [0–12] months. The number of RSV cases were divided by the population in the database for the same age group to compute a rate of cases per 1000 inhabitants.

### RSV season

One season was analyzed as only two years (2017 and 2018) were available in the database. An ample definition of RSV season was considered, from September 2017 to June 2018, considering, as a reference, the period in which national surveillance systems for influenza (which also report RSV tests, when performed) are in place, plus a margin of approximately one month. This period improves the capacity to detect potential RSV cases, considering the variability in the RSV seasonality across years and countries [16–18]. Furthermore, laboratory-confirmed RSV cases had been reported in September in the previous season (2016/17) in a Spanish region [19].

### Risk factors

Active diagnosis associated to the patients in the EMR during the analyzed period (chronical or acute pathologies) were used to identify children who had at least one medical condition regarded as a risk factor for RSV. The ICD-9-CM and ICD-10-ES diagnostic codes used to identify these risk factors (either as primary or related diagnostic) are detailed in the Additional file 1: Table S1. Risk factors such as heart disease, neuromuscular disorders, bronchopulmonary dysplasia, Down syndrome, immunodeficiency, congenital anomalies of respiratory system, congenital musculoskeletal anomalies, and cystic fibrosis were considered, based on expert advice. Patients were stratified by presence or absence of any of these risk factors/comorbidities. Premature birth, low weight or size at gestational age and exposure to tobacco were separately analyzed as potential risk factors as these are not underlying pathologies and may be less frequently inserted by medical coders.

### Resource utilization estimation

An RSV episode was defined as the day of physician visit (in any setting) when the index diagnosis was made (index date), together with a related period of 14 days before and 14 days after the date of the diagnosis, as performed in a similar study for a respiratory infection [20]. During this period, the healthcare resource consumption of patients with RSV was considered to be due to RSV, with the following exceptions: (i) for outpatient consultations only visits to a set of specialties more likely related to RSV symptoms or complications were considered, based on expert advice; (ii) for hospitalizations, only episodes with RSV as the primary or secondary diagnosis were included. In this case, the 14 days period after the diagnosis was applied to the date of the discharge of the patient.

Two aspects are considered in the analysis of resource utilization. On one hand, how many RSV patients visited at least once each of the healthcare setting during their RSV episode. Then, for those who visited the service/healthcare setting at least once, mean number of visits to each healthcare setting per RSV patient were computed, namely visits to primary care (PC), outpatient specialized care (OP), emergency department (ED) and hospital (HO), as inpatient.

The prescription of pharmaceutical products likely to be prescribed for RSV was also analyzed but was not included in the results as it represented a cost lower than 1%, since only retail prescription products are captured by the database.

### Cost estimation

The cost analysis was performed from the payer perspective and was based on resource utilization data combined with the unit cost of each resource. It comprised three steps and was stratified by age or by presence or absence of at least one risk factor.

### Mean healthcare cost per case per setting

Firstly, mean healthcare costs per RSV patient who visited each healthcare setting were computed. At this stage, the previously computed mean number of visits to each healthcare setting of each medically attended RSV patient, during their RSV period, were multiplied by the unit cost of each of those healthcare visits, to compute the mean cost per patient who visited each healthcare setting.

The mean cost per RSV case per setting were computed for PC, OP, ED and HO. By mean costs per case per setting, we mean, for instance, the mean cost of HO per RSV case amongst RSV cases who were hospitalized during their RSV episode.

### Mean healthcare cost per case

Then, these mean costs per case per setting were multiplied by the percentage of RSV cases in the database visiting each of the healthcare setting. As an example, the mean cost of HO per RSV case was obtained by multiplying the mean cost of HO amongst hospitalized RSV cases with the percentage of RSV cases who were hospitalized. The sum of the mean cost of PC, OP, ED and HO per RSV case results in the mean healthcare cost per RSV case.

### Total healthcare cost of RSV cases in the database

The mean healthcare costs per case per setting were multiplied by the number of RSV cases visiting each healthcare setting in the database.

### Unit costs of each resource

Unit cost per visit to each healthcare setting is not specific for RSV, except for hospitalization. Estimates of unit costs per type of healthcare visit were obtained from the eSalud Platform [21], considering official tariffs reported by autonomous communities, when available, and are detailed in Supporting Materials (Additional file 1: Table S2). For hospitalizations, the cost of each hospitalization observed in the database is individually estimated. The 3 M™ All Patient Refined Diagnosis Related Groups (APR DRGs) system (version 32) is used to calculate the degree of complexity for each hospitalization episode, considering variables related to the patient and episode. This is used to compute units of hospital production (UHP) for costing purposes [22, 23]. Estimated hospital mean cost per UHP is established for each hospital in the database considering the operating costs incurred by the hospital to carry out its activity to the production carried out by the hospital, measured through UHP. The average cost per UHP is updated annually through the IQVIA (former IASIST) Hospital TOP 20 Program.

## Results

### Studied population

Between September 2017 and June 2018, 3460 potential RSV cases were identified in the database in children aged < 5 years old, of which 257 (7.4%), 164 (4.7%), and 3,039 (87.8%) were coded with RSV-specific, unspecific bronchiolitis, and unspecific ALRI codes, respectively.

Medically attended cases per 1000 children were 25.2, 44.1 and 134.4 in children aged < 1 year, 3.6, 4.7 and 119.4 in children aged 2 years old, and 0.5, 0.6 and 35.3 in children aged between 2 and 5 years old, for RSV-specific, RSV-specific and Bronchiolitis and RSV-specific and ALRI codes, respectively. If only hospitalized cases were considered, these rates would be 25.0, 43.2 and 56.1 in children aged < 1 year, 3.6, 4.6 and 19.1 in children aged 2 years old, and 0.5, 0.5 and 3.6 in children aged between 2 and 5 years old, for RSV-specific, RSV-specific and Bronchiolitis and RSV-specific and ALRI cases, respectively. Table 2 reports the number of cases per 1000 inhabitants per age group, according to each RSV case definition, in total and for each visited healthcare setting only.

**Table 2:** Respiratory syncytial virus cases per 1000 children across settings of care, according to patients’ age (cases per 1000)

Months of age	RSV-specific					RSV-specific and bronchiolitis					RSV-specific and ALRI				
	Total	PC	OP	ED	HO	Total	PC	OP	ED	HO	Total	PC	OP	ED	HO
[0–1]	96.6	89.7	6.9	89.7	96.6	124.1	117.2	20.7	117.2	124.1	137.9	124.1	20.7	124.1	137.9
[1–2]	92.2	88.2	8.0	92.2	92.2	158.3	154.3	26.1	158.3	158.3	174.3	168.3	26.1	166.3	164.3
[2–3]	51.4	50.0	2.9	51.4	50.0	91.4	87.1	7.1	91.4	88.6	110.0	102.9	8.6	101.4	94.3
[3–6]	28.0	27.1	3.6	28.0	28.0	49.6	47.8	5.9	49.6	48.7	100.6	98.4	14.9	76.3	56.9
[6–12]	9.9	9.4	1.8	9.9	9.7	18.4	17.3	3.4	18.0	17.8	150.5	146.0	18.4	85.5	34.9
[0–12]	25.2	24.2	2.9	25.1	25.0	44.1	42.2	6.1	43.7	43.2	134.4	130.0	17.1	90.1	56.1
[12–24]	3.6	3.6	0.6	3.6	3.6	4.7	4.5	0.8	4.6	4.6	119.4	116.4	12.6	60.4	19.1
[24–36]	1.5	1.5	0.2	1.5	1.3	1.6	1.6	0.2	1.5	1.3	65.7	63.5	4.6	32.4	7.2
[36–60]	0.1	0.1	0.0	0.1	0.1	0.1	0.1	0.0	0.1	0.1	20.6	20.1	1.6	8.7	1.8
[0–24]	13.2	12.7	1.6	13.1	13.0	22.1	21.1	3.1	21.8	21.6	126.0	122.4	14.6	73.5	35.4
[24–60]	0.5	0.5	0.1	0.5	0.5	0.6	0.6	0.1	0.6	0.5	35.3	34.3	2.6	16.4	3.6
Total (< 60)	**5.0**	**4.9**	**0.6**	**5.0**	**4.9**	**8.2**	**7.9**	**1.2**	**8.1**	**8.0**	**67.5**	**65.5**	**6.8**	**36.6**	**14.8**

*ALRI* acute lower respiratory infection, *ED* emergency department, *HO* hospital (inpatient), *IR* incidence rate, *RSV* respiratory syncytial virus, *OP* outpatient (specialized care), *PC* primary care

### Seasonality

Similar seasonality patterns were observed across the distinct RSV definitions, with November being the month with more reported cases, namely: 33.2% in RSV-specific, 25.6% in RSV-specific and Bronchiolitis and 17.2% in RSV-specific and ALRI. The months of November to March (inclusive) concentrated 82.6%, 74.9% and 61.8% of RSV-specific, RSV-specific and Bronchiolitis and RSV-specific and ALRI cases, respectively.

### Demographics and clinical characteristics

The male to female ratio was 1.3, with 56.0% of RSV-specific and ALRI cases being observed in boys. Children aged < 1 year old accounted for 78.6%, 83.8% and 31.1% of RSV-specific, RSV-specific and Bronchiolitis and RSV-specific and ALRI cases, respectively. Amongst hospitalized cases, children aged < 1 year old accounted for 79.1%, 84.4% and 59.0% of RSV-specific, RSV-specific and Bronchiolitis and RSV-specific and ALRI cases, respectively. Children without any risk factor (i.e. otherwise healthy children) accounted for 87.9%, 87.9% and 93.1% of RSV-specific, RSV-specific and Bronchiolitis and RSV-specific and ALRI cases, respectively. Amongst the 6.9% of children presenting at least one risk factor for RSV in RSV-specific and ALRI cases, heart disease (2.9%) was the most frequently reported risk factor, followed by neuromuscular disorder (2.0%), congenital musculoskeletal anomalies (1.3%), congenital disorders of respiratory system (0.6%), Down syndrome (0.3%), immunodeficiency (0.2%), bronchopulmonary dysplasia (0.1%) and cystic fibrosis (0.1%). Prematurity was registered as a diagnosis in 1.2% of cases, low birth weight in 2.0% of cases and exposure to tobacco was not registered in any patient. Results for other case definitions, per age group, are detailed in Table 3.

**Table 3:** Percentage of respiratory syncytial virus patients, in total and by those visiting each healthcare setting, according to patients’ age or risk factors (% of cases)

Characteristics (age or risk factor)	RSV-specific					RSV-specific and bronchiolitis					RSV-specific and ALRI				
	Total	PC	OP	ED	HO	Total	PC	OP	ED	HO	Total	PC	OP	ED	HO
Male	55.6	55.0	50.0	55.5	56.5	59.4	59.4	65.0	59.4	60.5	56.0	55.9	64.3	55.8	58.3
Female	44.4	45.0	50.0	44.5	43.5	40.6	40.6	35.0	40.6	39.5	44.0	44.1	35.7	44.2	41.7
[0–1]	5.4	5.2	3.1	5.1	5.5	4.3	4.2	5.0	4.1	4.4	0.6	0.5	0.9	1.0	2.6
[1–2]	17.9	17.7	12.5	18.0	18.2	18.8	19.1	21.7	19.0	19.3	2.5	2.5	3.7	4.4	10.8
[2–3]	14.0	14.1	6.3	14.1	13.8	15.2	15.1	8.3	15.4	15.1	2.2	2.1	1.7	3.8	8.7
[3–6]	24.1	24.1	25.0	24.2	24.5	26.1	26.2	21.7	26.4	26.3	6.4	6.5	9.4	9.0	16.6
[6–12]	17.1	16.9	25.0	17.2	17.0	19.5	19.1	25.0	19.2	19.3	19.3	19.3	23.4	20.2	20.4
[0–12]	78.6	77.9	71.9	78.5	79.1	83.8	83.7	81.7	84.1	84.4	31.1	31.0	39.1	38.4	59.0
[12–24]	14.4	14.9	18.8	14.5	14.6	11.4	11.4	13.3	11.3	11.5	35.1	35.2	36.6	32.7	25.5
[24–36]	6.2	6.4	6.3	6.3	5.5	4.0	4.2	3.3	3.8	3.4	20.5	20.5	14.3	18.7	10.2
[36–60]	0.8	0.8	3.1	0.8	0.8	0.7	0.7	1.7	0.7	0.7	13.3	13.3	10.0	10.3	5.3
[0–24]	93.0	92.8	90.6	93.0	93.7	95.2	95.0	95.0	95.4	95.9	66.2	66.2	75.7	71.0	84.5
[24–60]	7.0	7.2	9.4	7.0	6.3	4.8	5.0	5.0	4.6	4.1	33.8	33.8	24.3	29.0	15.5
With risk factor	12.1	12.0	34.4	12.1	11.9	12.1	11.6	28.3	12.0	11.5	6.9	6.9	16.0	8.6	12.1
Without risk factor	87.9	88.0	65.6	87.9	88.1	87.9	88.4	71.7	88.0	88.5	93.1	93.1	84.0	91.4	87.9
Born preterm	4.3	3.6	12.5	3.9	4.3	4.3	3.7	10.0	4.1	4.4	1.2	1.1	4.6	1.9	3.0
Born with low weight/size	4.3	4.0	12.5	3.9	4.3	4.8	4.5	13.3	4.6	4.9	2.0	2.0	5.7	2.4	3.7

*ALRI* acute lower respiratory infection, *ED* emergency department, *HO* hospital (inpatient), *RSV* respiratory syncytial virus, *OP* outpatient (specialized care), *PC* primary care

### Resource utilization

#### RSV patients visiting each healthcare setting during their episode

Almost all RSV patients visited primary care (> 93%), regardless of the RSV definition and age group. The proportion of patients having an OP specialized care ranged from 10.1% in RSV-specific and ALRI to 14.3% in RSV-specific and Bronchiolitis. Most patients visited the ED, namely 99.6%, 98.8% and 54.3% of patients, in RSV-specific, RSV-specific and Bronchiolitis and RSV-specific and ALRI, respectively. The percentage of patients hospitalized for RSV was 98.4%, 97.4%, and 22.0% in RSV-specific, RSV-specific and Bronchiolitis and RSV-specific and ALRI, respectively.

On average, amongst RSV-specific and ALRI cases, 96.7% of children aged < 1 year old visited PC, 12.7% visited OP specialized care, 67.0% visited the ED and 41.7% were hospitalized for RSV.

Table 4 details the percentage of RSV patients who visited at least once each healthcare setting during their RSV episode, according to patients’ age and presence of risk factors, for each case definition.

**Table 4:** Percentage of respiratory syncytial virus patients visiting each healthcare setting during their episode, according to patients’ age or risk factors (% of cases visiting the service within cases observed in that age or risk factor group)

Characteristics (age or risk factor)	RSV-specific						RSV-specific and bronchiolitis					RSV-specific and ALRI					
	Total	PC	OP	ED	HO		Total	PC	OP	ED	HO	Total		PC	OP	ED	HO
[0–1]	100.0	92.9	7.1	92.9	100.0		100.0	94.4	16.7	94.4	100.0	100.0		90.0	15.0	90.0	100.0
[1–2]	100.0	95.7	8.7	100.0	100.0		100.0	97.5	16.5	100.0	100.0	100.0		96.6	14.9	95.4	94.3
[2–3]	100.0	97.2	5.6	100.0	97.2		100.0	95.3	7.8	100.0	96.9	100.0		93.5	7.8	92.2	85.7
[3–6]	100.0	96.8	12.9	100.0	100.0		100.0	96.4	11.8	100.0	98.2	100.0		97.8	14.8	75.8	56.5
[6–12]	100.0	95.5	18.2	100.0	97.7		100.0	93.9	18.3	97.6	96.3	100.0		97.0	12.3	56.8	23.2
[0–12]	100.0	96.0	11.4	99.5	99.0		100.0	95.8	13.9	99.2	98.0	100.0		96.7	12.7	67.0	41.7
[12–24]	100.0	100.0	16.2	100.0	100.0		100.0	95.8	16.7	97.9	97.9	100.0		97.4	10.5	50.6	16.0
[24–36]	100.0	100.0	12.5	100.0	87.5		100.0	100.0	11.8	94.1	82.4	100.0		96.6	7.0	49.4	11.0
[36–60]	100.0	100.0	50.0	100.0	100.0		100.0	100.0	33.3	100.0	100.0	100.0		97.6	7.6	42.0	8.7
[0–24]	100.0	96.7	12.1	99.6	99.2		100.0	95.8	14.2	99.0	98.0	100.0		97.1	11.6	58.3	28.1
[24–60]	100.0	100.0	16.7	100.0	88.9		100.0	100.0	15.0	95.0	85.0	100.0		97.0	7.3	46.5	10.1
With risk factor	100.0	96.8	35.5	100.0	96.8		100.0	92.2	33.3	98.0	92.2	100.0		97.1	23.4	67.8	38.5
Without risk factor	100.0	96.9	9.3	99.6	98.7		100.0	96.5	11.6	98.9	98.1	100.0		97.1	9.1	53.3	20.8
Born preterm	100.0	81.8	36.4	90.9	100.0		100.0	83.3	33.3	94.4	100.0	100.0		92.5	40.0	87.5	57.5
Born with low weight/size	100.0	90.9	36.4	90.9	100.0		100.0	90.0	40.0	95.0	100.0	100.0		95.7	29.0	66.7	40.6
Total	100.0	96.9	12.5	99.6	98.4		100.0	96.0	14.3	98.8	97.4	100.0		97.1	10.1	54.3	22.0

*ALRI* aute Lower Respiratory Infection,* ED* Emergency Department,* HO*Hospital (Inpatient),* OP* outpatient (specialized care),* PC* primary Care,,* RSV* respiratory syncytial virus

### Mean number of visits to each healthcare setting per visiting RSV patient

Amongst RSV-specific and ALRI cases with at least one visit to the healthcare setting, children aged < 1 year old had, on average, 9.3 visits to PC, 1.4 visits to OP specialized care, 2.3 visits to the ED and 1.2 hospitalizations for RSV. Mean visits per patient varied per age group but were relatively similar between patients who presented at least a risk factor compared to patients with no risk factor.

Table 5 presents the mean number of visits to each healthcare setting per RSV patient (amongst those who visited the healthcare setting at least once), according to patients’ age and presence of risk factors, for each case definition.

**Table 5:** Mean number of visits to each healthcare setting per respiratory syncytial virus patient visiting the healthcare setting at least once during their episode, according to patients’ age or risk factors (visits per patient*)

Characteristics (age or risk factor)	RSV-specific				RSV-specific and bronchiolitis				RSV-specific and ALRI			
	PC	OP	ED	HO	PC	OP	ED	HO	PC	OP	ED	HO
[0–1]	7.5	1.0	2.0	1.4	7.6	1.3	2.1	1.3	7.5	1.3	2.0	1.3
[1–2]	9.2	1.3	2.3	1.2	9.4	1.3	2.3	1.3	9.3	1.3	2.3	1.3
[2–3]	9.7	1.0	2.6	1.4	9.1	1.0	2.4	1.3	9.3	1.2	2.3	1.3
[3–6]	9.8	1.1	2.3	1.1	10.1	1.2	2.4	1.1	9.9	1.3	2.5	1.2
[6–12]	9.4	1.8	2.7	1.3	9.0	1.7	2.6	1.2	9.1	1.5	2.3	1.2
[0–12]	9.4	1.3	2.4	1.2	9.4	1.4	2.4	1.0	9.3	1.4	2.3	1.2
[12–24]	7.6	2.0	2.5	1.1	7.6	1.8	2.6	1.1	6.9	1.3	1.9	1.1
[24–36]	6.4	1.0	2.9	1.2	6.6	1.0	2.9	1.2	5.7	1.2	1.7	1.1
[36–60]	6.5	1.0	3.0	2.0	7.0	1.0	2.7	1.7	5.3	1.4	1.8	1.2
[0–24]	9.1	1.5	2.4	1.2	9.2	1.4	2.4	1.2	8.0	1.3	2.1	1.2
[24–60]	6.4	1.0	2.9	1.3	6.7	1.0	2.9	1.3	5.5	1.3	1.8	1.1
With risk factor	9.6	1.9	2.5	1.3	9.2	1.8	2.4	1.3	7.6	1.6	2.2	1.3
Without risk factor	8.8	1.2	2.5	1.2	9.0	1.2	2.5	1.2	7.1	1.3	2.0	1.2
Born preterm	16.1	1.5	1.8	1.1	13.1	1.7	1.8	1.1	10.3	1.4	2.0	1.0
Born with low weight /size	13.4	1.5	2.3	1.3	10.9	1.8	2.2	1.3	9.0	1.6	2.1	1.2
Total	8.9	1.4	2.5	1.2	9.0	1.4	2.5	1.2	7.2	1.3	2.0	1.2

*ALRI *acute lower respiratory infection, *ED* emergency department, *HO* Hospital (inpatient), *OP* outpatient (specialized care), *PC* primary care, *RSV* respiratory syncytial virus

*Each patient may then visit each healthcare setting more than once, even in the same day (e.g. visit to general practitioner and visit to nurse in the same primary care center)

### Direct healthcare cost

#### Mean healthcare cost per case per setting

Amongst RSV-specific and ALRI cases in children aged < 1 year old, each child visiting PC generated a mean healthcare cost of PC visits of €486; mean cost of OP visits per RSV patient seeking OP care was estimated at €270; mean cost of ED visits per RSV patient visiting the ED was estimated at €408; and mean cost of hospitalization per hospitalized RSV patient was estimated at €2,335. These are mean costs per medically attended RSV patient and may include more than one visit per patient. These costs are detailed in Table 6 for each RSV definition and according to patients’ age and presence of risk factors.

**Table 6:** Mean direct healthcare costs of visits to each healthcare setting per respiratory syncytial virus patient who visited each healthcare setting and weighted total mean direct healthcare cost per respiratory syncytial virus patient, according to patients’ age or risk factors (€ per patient)

Characteristics (age or risk factor)	RSV-specific					RSV-specific and bronchiolitis					RSV-specific and ALRI				
	Total	PC	OP	ED	HO	Total	PC	OP	ED	HO	Total	PC	OP	ED	HO
[0–1]	5227	388	223	348	4527	4870	396	265	358	4114	6629	392	265	348	5923
[1–2]	3483	470	254	393	2617	3524	471	262	405	2617	3334	467	262	403	2610
[2–3]	3297	511	223	450	2405	2907	475	223	419	2083	2628	489	244	407	2073
[3–6]	2761	490	239	407	1850	2651	513	252	416	1742	1912	511	257	428	1858
[6–12]	3540	484	316	467	2613	2971	471	306	459	2102	1248	483	278	404	2225
[0–12]	3362	481	266	421	2475	3080	481	269	421	2209	1753	486	270	408	2335
[12–24]	3252	408	347	442	2346	2938	405	316	444	2106	896	374	247	327	2128
[24–36]	3202	359	223	511	2633	3044	369	223	511	2633	704	313	242	297	2169
[36–60]	6009	341	223	522	5034	4625	398	223	464	3689	650	289	273	318	2443
[0–24]	3345	470	283	424	2455	3063	472	276	424	2196	1299	427	259	371	2273
[24–60]	3514	357	223	512	2933	3282	373	223	504	2819	683	304	255	305	2262
With risk factor	3873	478	336	432	2954	3923	466	325	418	3228	2065	404	292	387	3486
Without risk factor	3286	459	247	430	2422	2957	467	252	429	2092	1018	384	251	348	2104
Born preterm	5121	744	285	313	4124	4720	630	306	317	3793	2785	517	278	353	3281
Born with low weight/size	5793	623	285	400	4759	5321	534	316	385	4349	2213	459	291	371	3554
Total	3357	461	278	430	2485	3074	467	273	427	2222	1091	385	258	352	2271

*ALRI* acute lower respiratory infection, *ED* emergency department, *HO* hospital (inpatient), *OP* outpatient (specialized care), *PC* primary care, *RSV* respiratory syncytial virus

### Mean healthcare cost per case

**Fig. 2:**
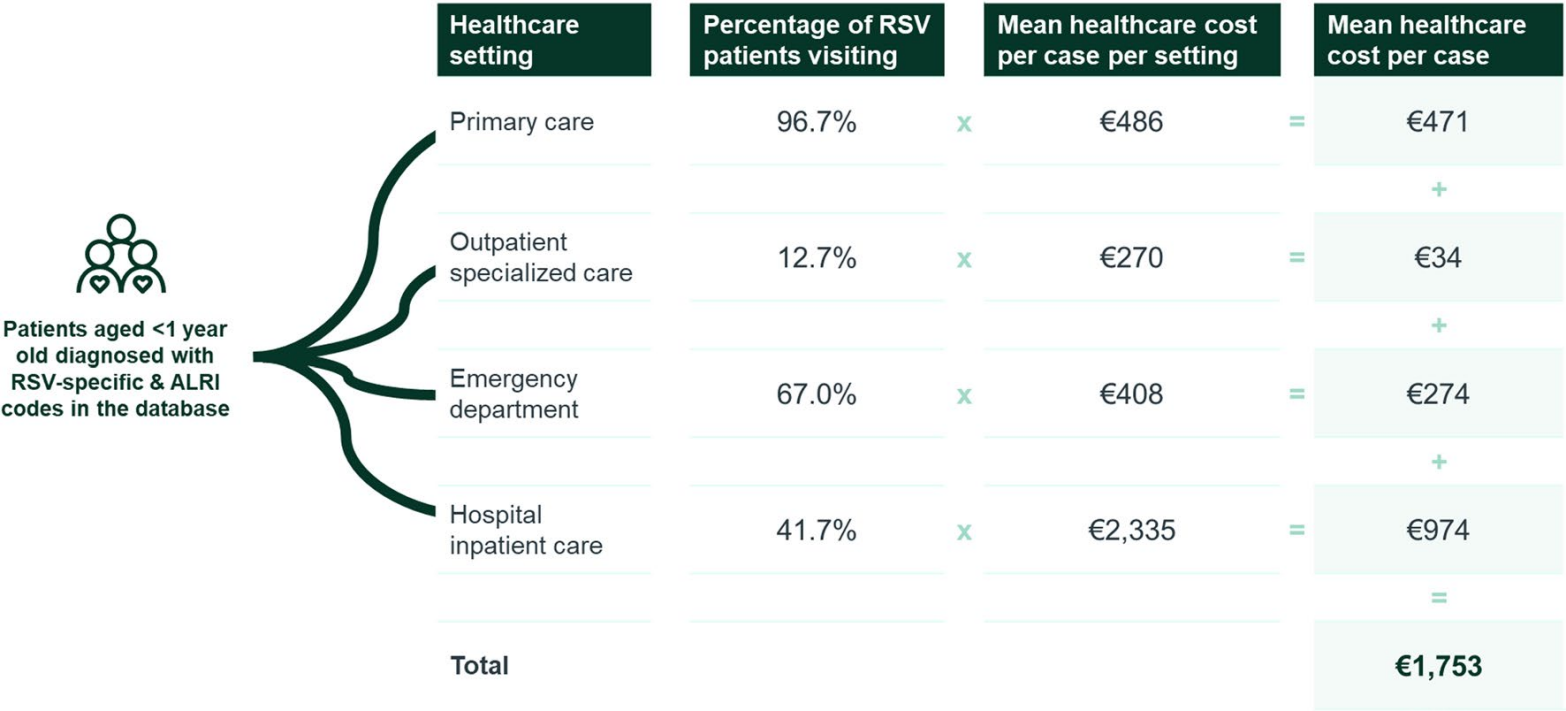
Mean healthcare cost per RSV-specific and ALRI case aged bellow one year old

Combining the mean healthcare costs per case per setting with the previously stated percentage of children aged < 1 year old visiting each healthcare setting, results in a mean healthcare cost per medically attended RSV-specific and ALRI case of €1753 in the first year of life (Fig. 2). This mean cost decreased to €896 in children aged two years old, and €683 between 2 and 5 years old. These costs are detailed in Table 6 for each RSV definition and according to patients’ age and presence of risk factors.

### Total healthcare cost of medically attended RSV patients

In season 2017/18, the 3460 children aged < 5 years old who have been diagnosed with RSV-specific and ALRI codes in the two Spanish regions included in the study have cost €3.8 Million to the NHS, mainly driven by hospitalizations (45.8%, Table 7). Most of the healthcare costs were generated by children aged < 1 year old, who contributed to 78.7%, 84.0% and 50.0% of the healthcare costs of medically attended cases, for RSV-specific, RSV-specific and Bronchiolitis and RSV-specific and ALRI cases, respectively (Table 8). Most costs were generated by otherwise healthy children, regardless of age, who accounted for 86.1%, 84.5% and 86.9% of the direct healthcare costs of medically attended cases, for RSV-specific, RSV-specific and Bronchiolitis and RSV-specific and ALRI cases, respectively.

**Table 7:** Contribution of each healthcare setting to the total direct healthcare cost of medically attended respiratory syncytial virus patients, according to patients’ age or risk factors (% of total cost observed in that age or risk factor group)

Characteristics (age or risk factor)	RSV-specific						RSV-specific and bronchiolitis					RSV-specific and ALRI					
	Total	PC	OP	ED	HO		Total	PC	OP	ED	HO	Total		PC	OP	ED	HO
[0–1]	100.0	6.9	0.3	6.2	86.6		100.0	7.7	0.9	6.9	84.5	100.0		5.3	0.6	4.7	89.4
[1–2]	100.0	12.9	0.6	11.3	75.1		100.0	13.0	1.2	11.5	74.3	100.0		13.5	1.2	11.5	73.8
[2–3]	100.0	15.1	0.4	13.6	70.9		100.0	15.6	0.6	14.4	69.4	100.0		17.4	0.7	14.3	67.6
[3–6]	100.0	17.2	1.1	14.7	67.0		100.0	18.7	1.1	15.7	64.5	100.0		26.1	2.0	17.0	54.9
[6–12]	100.0	13.1	1.6	13.2	72.1		100.0	14.9	1.9	15.1	68.2	100.0		37.6	2.7	18.4	41.3
[0–12]	100.0	13.8	0.9	12.5	72.9		100.0	15.0	1.2	13.6	70.3	100.0		26.8	2.0	15.6	55.6
[12–24]	100.0	12.5	1.7	13.6	72.1		100.0	13.2	1.8	14.8	70.2	100.0		40.7	2.9	18.4	38.0
[24–36]	100.0	11.2	0.9	16.0	71.9		100.0	12.1	0.9	15.8	71.2	100.0		43.0	2.4	20.8	33.8
[36–60]	100.0	5.7	1.9	8.7	83.8		100.0	8.6	1.6	10.0	79.8	100.0		43.5	3.2	20.6	32.8
[0–24]	100.0	13.6	1.0	12.6	72.8		100.0	14.8	1.3	13.7	70.3	100.0		31.9	2.3	16.6	49.1
[24–60]	100.0	10.2	1.1	14.6	74.2		100.0	11.4	1.0	14.6	73.0	100.0		43.1	2.7	20.7	33.4
With risk factor	100.0	11.9	3.1	11.2	73.8		100.0	11.0	2.8	10.4	75.8	100.0		19.0	3.3	12.7	65.0
Without risk factor	100.0	13.5	0.7	13.0	72.7		100.0	15.2	1.0	14.3	69.4	100.0		36.6	2.3	18.2	42.9
Born preterm	100.0	11.9	2.0	5.6	80.5		100.0	11.1	2.2	6.3	80.4	100.0		17.2	4.0	11.1	67.7
Born with low weight/size	100.0	9.8	1.8	6.3	82.2		100.0	9.0	2.4	6.9	81.7	100.0		19.9	3.8	11.2	65.2
Total	100.0	13.3	1.0	12.8	72.9		100.0	14.6	1.3	13.7	70.4	100.0		34.3	2.4	17.5	45.8

*ALRI* acute lower respiratory infection, *ED* emergency department, *HO* hospital (inpatient), *RSV* respiratory syncytial virus, *OP* outpatient (specialized care), *PC* primary care

**Table 8:** Percentage of total direct healthcare cost of medically attended respiratory syncytial virus patients, generated by each group of patients’, according to their gender, age, and presence of risk factors (%)

Characteristics (age or risk factor)	RSV-specific					RSV-specific and bronchiolitis					RSV-specific and ALRI				
	Total	PC	OP	ED	HO	Total	PC	OP	ED	HO	Total	PC	OP	ED	HO
Male	52.7	53.4	54.2	52.3	52.6	58.9	59.9	66.1	58.1	58.8	58.1	57.1	65.5	56.6	59.0
Female	47.3	46.6	45.8	47.7	47.4	41.1	40.1	33.9	41.9	41.2	41.9	42.9	34.5	43.4	41.0
[0–1]	8.5	4.4	2.5	4.1	10.1	6.8	3.6	4.8	3.4	8.1	3.5	0.5	0.9	0.9	6.9
[1–2]	18.6	18.0	11.5	16.4	19.1	21.5	19.2	20.8	18.0	22.7	7.7	3.0	3.8	5.1	12.4
[2–3]	13.8	15.6	5.0	14.7	13.4	14.4	15.4	6.8	15.1	14.2	5.4	2.7	1.6	4.4	7.9
[3–6]	19.8	25.6	21.5	22.9	18.2	22.5	28.8	20.0	25.7	20.7	11.3	8.6	9.4	11.0	13.5
[6–12]	18.1	17.7	28.5	18.6	17.9	18.8	19.2	28.0	20.6	18.2	22.1	24.2	25.3	23.2	20.0
[0–12]	78.7	81.3	69.0	76.8	78.7	84.0	86.2	80.5	82.9	83.9	50.0	39.1	41.0	44.5	60.7
[12–24]	13.9	13.1	23.5	14.8	13.8	10.9	9.9	15.4	11.7	10.9	28.8	34.2	35.0	30.4	23.9
[24–36]	5.9	5.0	5.0	7.4	5.9	4.0	3.3	2.7	4.6	4.0	13.3	16.6	13.4	15.8	9.8
[36–60]	1.4	0.6	2.5	0.9	1.6	1.1	0.6	1.4	0.8	1.2	7.9	10.0	10.6	9.3	5.7
[0–24]	92.7	94.4	92.5	91.6	92.5	94.9	96.0	95.9	94.6	94.7	78.8	73.4	76.0	74.9	84.6
[24–60]	7.3	5.6	7.5	8.4	7.5	5.1	4.0	4.1	5.4	5.3	21.2	26.6	24.0	25.1	15.4
With risk factor	13.9	12.5	41.6	12.2	14.1	15.5	11.6	33.8	11.7	16.7	13.1	7.3	18.2	9.5	18.6
Without risk factor	86.1	87.5	58.4	87.8	85.9	84.5	88.4	66.2	88.3	83.3	86.9	92.7	81.8	90.5	81.4
Born preterm	6.5	5.8	12.8	2.8	7.2	6.6	5.0	11.2	3.0	7.5	3.0	1.5	4.9	1.9	4.4
Born with low weight/size	7.4	5.4	12.8	3.6	8.3	8.2	5.1	15.4	4.1	9.5	4.0	2.3	6.5	2.6	5.8

*ALRI* acute lower respiratory infection, *ED* emergency department, *HO* hospital (inpatient), *RSV* respiratory syncytial virus, *OP* outpatient (specialized care), *PC* primary care

Table 7 details the contribution of the costs with each healthcare setting to the total direct healthcare cost of medically attended RSV patients (in percentage) for each RSV definition and according to patients’ age and presence of risk factors. Table 8 details the percentage of the total healthcare cost of medically attended RSV patients, generated by each group of patients’, according to their gender, age, and presence of risk factors, for each case definition.

## Discussion

A high clinical and economic burden of medically attended ALRI cases potentially related to RSV occurs in children aged < 5 years old. To our knowledge, this is the first study reporting the burden of medically attended ALRI cases potentially related to RSV in Spanish children, considering distinct RSV definitions and healthcare settings. Using an EMR based database which includes all visits of the population from two Spanish regions to the NHS, for all healthcare settings, the study provides evidence on the burden of potential RSV disease in children < 5 years, beyond hospitalization. Such data is currently scarce in Europe as studies focus mostly on hospitalizations and it is vital for the definition of evidence-based preventive policies.

As expected, the need for medical care was particularly intense during the first year of life. These findings are consistent with results from other studies from western countries using similar methodologies for RSV-specific and unspecified acute bronchiolitis case [7, 24–26]. Incidence rates markedly decrease as age increases—as previously reported [13–15, 27]—being 20 times (RSV-Specific) and 28 times (RSV-Specific and Bronchiolitis) higher in children aged < 1 year old than in those aged between 1 and 5 years old.

Most cases were observed between November and March (61.8–82.6%, depending on the definition), as expected based on previous studies [16, 19]. The peak of cases was observed in November, as had also been reported for Galicia in the previous season [19].

Children who had a risk factor for RSV were 1.9 times more likely to be hospitalized for RSV-specific and ALRI (38.5% vs. 20.8% in those without a risk factor). No relevant differences were observed in the other case definitions as, regardless of presence of risk factors, almost all children were hospitalized for RSV (> 92%). The mean direct healthcare cost per medically attended potential RSV case also increased when a risk factor was present, which is coherent with published evidence that children with underlying medical conditions such as congenital heart disease, and chronic pulmonary and cardiovascular diseases, and prematurity, are more vulnerable to severe RSV infection [28–30]. However, even considering diagnosis received both in inpatient and outpatient setting to identify patients’ concurrent medical conditions, most identified RSV cases were observed in otherwise healthy children (87.9–93.1%, according to the RSV definition). This is consistent with previous national and international studies [6, 31]. A study on RSV bronchiolitis hospitalizations in Spain between 1997 and 2011 had identified 3.2% of cases with comorbidities, in those aged < 5 years old [29].

Three case definitions were used with most differences being observed between RSV-specific and ALRI and the other two (RSV-specific and RSV-specific and Bronchiolitis). Data appears to suggest that the latter definitions are mostly used for severe cases, as 99% of patients receiving these diagnoses visited the emergency services and over 97% have been hospitalized, versus 54% and 22% in RSV-specific and ALRI definition, respectively. This holds true even if only cases during the first year of life are considered. The proportion of cases generated by children aged < 1 year old also decreases as RSV definitions become “broader”, which might be a reflex of less frequent RSV testing and coding as age increases. In 2009, a multicenter Spanish study including 5,647 children < 2 years old with a first acute bronchiolitis episode – of which 51.2% proceeding from the emergency department, 28.9% from hospitalizations, 18.3% from primary care and 1.6% from ICU - has reported that RSV diagnosis tests were performed in only 37.4% of acute bronchiolitis medically attended cases, of which 63.5% were RSV-positive and found that younger age was associated with increased likelihood of being tested for RSV.[32] An international study has also reported decreased testing among children aged above six months [27]. Other authors have also reported that RSV-positive cases are more frequently diagnosed as bronchiolitis in patients aged < 1 year old while in children aged between 2 and 5 years old, pneumonia was more frequent [7]. Overall, evidence suggests a potential age-associated bias in the diagnosis of RSV in children admitted for RSV-associated respiratory disease, supported also by the uncommon diagnosis of RSV-associated pneumonia [14, 33, 34].

The present study highlighted the magnitude of potentially underestimated RSV cases, especially using RSV-specific codes. Cases per 1000 were 1.6 and 13.5 times higher using the RSV-specific and Bronchiolitis and the RSV-specific and ALRI definition, respectively, than using RSV-specific codes alone. In hospitalized cases per 1000 children the increase was the same for RSV-specific and Bronchiolitis but only 1.9 for RSV-specific and ALRI. Results appear to support previous findings that combining RSV-specific with unspecified ALRI ICD diagnosis may indeed help to better understand the burden of RSV, particularly if testing is not systematically performed in a specific healthcare setting or region [1, 11, 12]. In the U.S., Hall et al. had also found a greater RSV diagnosis capacity at the hospitals, reporting that only 3% of outpatients with RSV infection received the diagnosis of RSV-associated illness, as compared with 45% of inpatients [7].

We reported rates of 8.0 hospitalized children per 1,000 children aged < 5 years old using RSV-specific and Bronchiolitis and 21.6 in those aged < 2 years old, which are similar to those reported by Gil-Prieto et al. for Spain, namely of 10.7 and 24.1 per 1,000 children aged bellow 5 and 2 years old, respectively, during 1997–2011 [29]. In Valencia, a population study on 198,223 children born between 2009 and 2012, has reported an incidence rate of bronchiolitis (all causes) of 164 cases per 1000 children aged < 2 years old, which was 87% lower if only hospitalized cases were considered (21 per 1000) [13]. Amongst those hospitalized, 57.6% had either an RSV ICD-9 diagnosis or had a positive RSV test result [13]. Assuming that the same rate of RSV-cases coded as bronchiolitis could be observed in the outpatient setting this would result in an incidence rate of approximately 94 RSV cases per 1000 children, which is closer to the one obtained by our RSV-specific and ALRI definition (126 per 1000) than in RSV-Specific and Bronchiolitis (22 per 1000). This difference might reflect regional variations in testing and coding practices, but, in both cases, reflects a high burden of acute respiratory infections in Spanish children that needs to be better understood and addressed. Further studies combining health administrative data and RSV laboratory data for Spain to assess the validity of each diagnosis code in Spain would be of interest, although regional variation must be accounted for.

An important limitation of this study is that it does not enable to quantify the proportion of the reported unspecified ALRI cases that have been caused by RSV, and not by other infectious agents, which may unduly inflate the estimates calculated under the definition RSV-specific and ALRI. While several studies name RSV as the most common pathogen identified in young children with ALRI (mainly pneumonia and bronchiolitis) [1, 35, 36], there are other infectious agents that can cause severe ALRI— such as pneumonia—including viruses, bacteria and fungi [35, 37] In children, bacterial pneumonia is most often caused by Streptococcus pneumoniae (pneumococcus) or by Haemophilus influenzae type b (Hib) while RSV is the most common viral cause of pneumonia [35, 37], with a role that is expected to increase as the use of vaccines against the most common types of bacterial pneumonia grows [38–40], as well as the capacity to detect virus through molecular diagnostic, as differentiating between viral and bacterial pneumonias radiographically is difficult [35]. A systematic review and meta-analysis performed by Shi et al. (2015) found evidence supporting that RSV, influenza (IFV), parainfluenza (PIV), human metapneumovirus (HMPV), and rhinovirus (RV) are important causes of ALRI in young children, estimating the percentage of (severe) ALRI which could be attributed to each virus to be 90%, 80%, 70%, 73% and 30%, respectively [41].

However, few studies on pneumonia etiology were conducted in high-income countries [40]. A recent prospective case–control study performed in West Australia has estimated the contribution of respiratory viruses and bacteria to pneumonia in people aged < 18 years. The study reported that RSV had the highest contribution amongst detectable respiratory viruses responsible for the analyzed pneumonias requiring hospitalization and concluded that RSV and HMPV are probable pneumonia-causing pathogens in children with pneumonia [40]. It is thus reasonable to expect that, while this broader RSV-specific and ALRI definition may have the benefit of potentially capturing more cases of RSV caused ALRI, it may also have the pitfall of potentially including non-RSV caused ALRI [11, 12].

In either case, findings from the BARI study support that RSV is a major driver of demand of healthcare resources at all levels of care in children, resulting in important costs to the NHS. Hospitalization rates were higher in children bellow 2 years old. In our study, children aged bellow 2 years old accounted for 78.8–94.9% of total direct healthcare costs of medically attended RSV patients aged bellow 5 years old, and 84.6–94.7% of hospitalization costs, which is consistent with previous findings for Spain [29]. An important part of the cost was driven by children aged bellow 1 year old, who accounted for 50.0–84.0% of total direct healthcare costs of medically attended RSV patients aged bellow 5 years old, and 60.7–83.9% of hospitalization costs. Otherwise healthy children generated most of the NHS costs (84.5–86.9%, according to the RSV definition). Mean direct healthcare cost per patient with RSV-specific diagnosis was higher, given the greater proportion of patients visiting the emergency department or being hospitalized, namely €3,357, €3,345, and €3,362 in patients aged < 5, <2 and < 1 year old, respectively.

Still, RSV burden is expected to be even higher if other factors are considered. A high frequency of primary care visits during the 28 days around the RSV diagnosis was found in our study, suggesting not only a burden for the healthcare system but potentially relevant productivity losses from the children’s parents, which were not quantified in our study. Importantly, children having severe RSV infection are more likely to develop respiratory complications such as wheezing and asthma, generating a long term burden for those infected [1, 5, 10]. A study found that premature infants infected by RSV had almost twice the number of primary care visits than controls during the two years following the RSV infection.[42] An important limitation of our study was that this long-lasting burden of the RSV-infection was not studied.

The major limitations of the present study are that it is based on a population sample and that it relies on data from an EMR database without information of whether RSV laboratory testing was performed. Data is subject to coding errors or missing information. Due to the low precision of RSV codification in primary care the studied population may be biased towards more severe RSV cases. The method used to estimate resource utilization during the RSV period may overestimate the number of visits due to RSV, as some could have been performed, regardless of RSV diagnosis. As previously mentioned, the study does not consider healthcare resources consumed in a private healthcare setting, nor indirect costs related to productivity losses or long-term burden. Importantly, the database is not aimed to be nationally representative as population from only two regions was used. Furthermore, this study was performed only during one RSV season, which can limit comparisons with results from other studies as RSV is a seasonal disease that commonly displays annual or biennial seasonality.

## Conclusions

RSV is a major driver of demand for medical care by Spanish children. The burden of RSV is particularly high in children under 1 year of age. Most cases and direct healthcare costs were observed in otherwise healthy children born at term. Analyzing only hospitalizations and RSV-specific diagnosis is expected to substantially underestimate the burden of the disease to the NHS. These findings highlight the need to improve RSV testing and surveillance, and to adopt preventive solutions that enable protection of all infants, particularly during the first year of life.

## Supplementary Information

**Additional file 1: Table S1**. List of analyzed risk factors andICD9/10 codes used. **Table S2. **Unit costs considered for each healthcare visit.

## Data Availability

The data that support the findings of this study are available from IQVIA, but restrictions apply to the availability of these data, which were used under license for the current study, and so are not publicly available. Data are however available from the authors upon reasonable request and with permission of IQVIA. Those wishing to request the data from this study should contact the author Mafalda Carmo.
