# Supplementary material for: Clinical and economic burden of respiratory syncytial virus in Spanish children: the BARI study
Source: BMC Infect Dis. 2022 Sep 29;22:759. doi: 10.1186/s12879-022-07745-0 (PMC9520861; doi:10.1186/s12879-022-07745-0)
Supplement: Supplementary file 1 — Additional file 1: Table S1. List of analyzed risk factors andICD9/10 codes used. Table S2. Unit costs considered for each healthcare visit. [file 12879_2022_7745_MOESM1_ESM.docx]

## Supplementary Materials

**Table S1 List of analyzed risk factors and ICD9/10 codes used**

| **Risk factor** | **Description** | **ICD9** | **ICD10** |
| --- | --- | --- | --- |
| **Risk factors not related to underlying medical diseases** | | | |
| **Preterm** | Extreme immaturity of newborn, unspecified weeks of gestation | 765.20 | P07.20 |
|  | Extreme immaturity of newborn, gestational age less than 23 completed weeks | 765.21 | P07.21 |
|  | Extreme immaturity of newborn, gestational age 23 completed weeks | 765.21 | P07.22 |
|  | Extreme immaturity of newborn, gestational age 24 completed weeks | 765.22 | P07.23 |
|  | Extreme immaturity of newborn, gestational age 25 completed weeks | 765.23 | P07.24 |
|  | Extreme immaturity of newborn, gestational age 26 completed weeks | 765.23 | P07.25 |
|  | Extreme immaturity of newborn, gestational age 27 completed weeks | 765.24 | P07.26 |
|  | Preterm [premature] newborn [other] | - | P07.3 |
|  | Preterm newborn, unspecified weeks of gestation | 765.09; 765.19; 765.20 | P07.30 |
|  | Preterm newborn, gestational age 28 completed weeks | 765.24 | P07.31 |
|  | Preterm newborn, gestational age 29 completed weeks | 765.25 | P07.32 |
|  | Preterm newborn, gestational age 30 completed weeks | 765.25 | P07.33 |
|  | Preterm newborn, gestational age 31 completed weeks | 765.26 | P07.34 |
|  | Preterm newborn, gestational age 32 completed weeks | 765.26 | P07.35 |
|  | Preterm newborn, gestational age 33 completed weeks | 765.27 | P07.36 |
|  | Preterm newborn, gestational age 34 completed weeks | 765.27 | P07.37 |
|  | Preterm newborn, gestational age 35 completed weeks | 765.28 | P07.38 |
|  | Preterm newborn, gestational age 36 completed weeks | 765.28 | P07.39 |
| **Low weight or size at gestational age** | Newborn light for gestational age, unspecified weight | 764.00 | P05.00 |
|  | Newborn light for gestational age, less than 500 grams | 764.01 | P05.01 |
|  | Newborn light for gestational age, 500-749 grams | 764.02 | P05.02 |
|  | Newborn light for gestational age, 750-999 grams | 764.03 | P05.03 |
|  | Newborn light for gestational age, 1000-1249 grams | 764.04 | P05.04 |
|  | Newborn light for gestational age, 1250-1499 grams | 764.05 | P05.05 |
|  | Newborn light for gestational age, 1500-1749 grams | 764.06 | P05.06 |
|  | Newborn light for gestational age, 1750-1999 grams | 764.07 | P05.07 |
|  | Newborn light for gestational age, 2000-2499 grams | 764.08 | P05.08 |
|  | Newborn light for gestational age, 2500 grams and over | 764.09 | P05.09 |
|  | Newborn small for gestational age, unspecified weight | 764.10 | P05.10 |
|  | Newborn small for gestational age, less than 500 grams | 764.11 | P05.11 |
|  | Newborn small for gestational age, 500-749 grams | 764.12 | P05.12 |
|  | Newborn small for gestational age, 750-999 grams | 764.13 | P05.13 |
|  | Newborn small for gestational age, 1000-1249 grams | 764.14 | P05.14 |
|  | Newborn small for gestational age, 1250-1499 grams | 764.15 | P05.15 |
|  | Newborn small for gestational age, 1500-1749 grams | 764.16 | P05.16 |
|  | Newborn small for gestational age, 1750-1999 grams | 764.17 | P05.17 |
|  | Newborn small for gestational age, 2000-2499 grams | 764.18 | P05.18 |
|  | Newborn small for gestational age, other | 764.19 | P05.19 |
|  | Newborn affected by fetal (intrauterine) malnutrition not light or small for gestational age | 764.20 | P05.2 |
|  | Newborn affected by slow intrauterine growth, unspecified | 764.90 | P05.9 |
|  | Extremely low birth weight newborn, unspecified weight | 765.0 765.10 | P07.00 |
|  | Extremely low birth weight newborn, less than 500 grams | 765.01765.11 | P07.01 |
|  | Extremely low birth weight newborn, 500-749 grams | 765.02  765.12 | P07.02 |
|  | Extremely low birth weight newborn, 750-999 grams | 765.03  765.13 | P07.03 |
|  | Other low birth weight newborn, unspecified weight | 765.00  765.10 | P07.10 |
|  | Other low birth weight newborn, 1000-1249 grams | 765.04 765.14 | P07.14 |
|  | Other low birth weight newborn, 1250-1499 grams | 765.05  765.15 | P07.15 |
|  | Other low birth weight newborn, 1500-1749 grams | 765.06  765.16 | P07.16 |
|  | Other low birth weight newborn, 1750-1999 grams | 765.07  765.17 | P07.17 |
|  | Other low birth weight newborn, 2000-2499 grams | 765.08  765.18 | P07.18 |
| **Exposure to tobacco** | Exposure to (parental) (environmental) tobacco smoke in the perinatal period | 760.8 | P96.81 |
|  | Contact with and (suspected) exposure to environmental tobacco smoke (acute) (chronic) | V15.89; E869.4 | Z77.22 |
| **Risk factors related to underlying medical diseases** | | | |
| **Heart disease** | Other chronic pulmonary heart diseases | 416.8 | I27.89; I27.2 |
|  | Other primary cardiomyopathies | 425.4 | I42.5; I42.8 |
|  | Congenital malformations of cardiac chambers and connections |  | Q20 |
|  | Common arterial trunk | 745.0 | Q20.0 |
|  | Double outlet right ventricle | 745.11 | Q20.1 |
|  | Double outlet left ventricle | 745.19 | Q20.2 |
|  | Discordant ventriculoarterial connection | 745.10; 745.19 | Q20.3 |
|  | Double inlet ventricle | 745.3 | Q20.4 |
|  | Discordant atrioventricular connection | 745.12 | Q20.5 |
|  | Isomerism of atrial appendages | 745.8 | Q20.6 |
|  | Other congenital malformations of cardiac chambers and connections | 745.19; 745.7; 745.8 | Q20.8 |
|  | Congenital malformation of cardiac chambers and connections, unspecified | 746.9 | Q20.9 |
|  | Congenital malformations of cardiac septa |  | Q21 |
|  | Ventricular septal defect | 745.4 | Q21.0 |
|  | Atrial septal defect | 745.5 | Q21.1 |
|  | Atrioventricular septal defect | 745.60; 745.61; 745.69 | Q21.2 |
|  | Tetralogy of Fallot | 745.2 | Q21.3 |
|  | Aortopulmonary septal defect | 745.8 | Q21.4 |
|  | Other congenital malformations of cardiac septa | 745.8 | Q21.8 |
|  | Congenital malformation of cardiac septum, unspecified | 745.9 | Q21.9 |
|  | Congenital malformations of pulmonary and tricuspid valves |  | Q22 |
|  | Pulmonary valve atresia | 746.01 | Q22.0 |
|  | Congenital pulmonary valve stenosis | 746.02 | Q22.1 |
|  | Congenital pulmonary valve insufficiency | 746.09 | Q22.2 |
|  | Other congenital malformations of pulmonary valve | 746.00 | Q22.3 |
|  | Congenital tricuspid stenosis | 746.1 | Q22.4 |
|  | Ebstein's anomaly | 746.2 | Q22.5 |
|  | Hypoplastic right heart syndrome | 746.1 | Q22.6 |
|  | Other congenital malformations of tricuspid valve | 746.1 | Q22.8 |
|  | Congenital malformation of tricuspid valve, unspecified | 746.1 | Q22.9 |
|  | Congenital malformations of aortic and mitral valves |  | Q23 |
|  | Congenital stenosis of aortic valve | 746.3 | Q23.0 |
|  | Congenital insufficiency of aortic valve | 746.4 | Q23.1 |
|  | Congenital mitral stenosis | 746.5 | Q23.2 |
|  | Congenital mitral insufficiency | 746.6 | Q23.3 |
|  | Hypoplastic left heart syndrome | 746.7 | Q23.4 |
|  | Other congenital malformations of aortic and mitral valves | 746.89 | Q23.8 |
|  | Congenital malformation of aortic and mitral valves, unspecified | 746.89 | Q23.9 |
|  | Other congenital malformations of heart |  | Q24 |
|  | Dextrocardia | 746.87 | Q24.0 |
|  | Levocardia | 746.87 | Q24.1 |
|  | Cor triatriatum | 746.82 | Q24.2 |
|  | Pulmonary infundibular stenosis | 746.83 | Q24.3 |
|  | Congenital subaortic stenosis | 746.81 | Q24.4 |
|  | Malformation of coronary vessels | 746.85 | Q24.5 |
|  | Congenital heart block | 746.86 | Q24.6 |
|  | Other specified congenital malformations of heart | 746.84; 746.87; 746.89 | Q24.8 |
|  | Congenital malformation of heart, unspecified | 746.9 | Q24.9 |
|  | Congenital malformations of great arteries |  | Q25 |
|  | Patent ductus arteriosus | 747.0 | Q25.0 |
|  | Coarctation of aorta | 747.10 | Q25.1 |
|  | Atresia of aorta | 747.11; 747.22 | Q25.2 |
|  | Interruption of aortic arch |  | Q25.21 |
|  | Other atresia of aorta |  | Q25.29 |
|  | Supravalvular aortic stenosis | 747.22 | Q25.3 |
|  | Other congenital malformations of aorta | 747.20; 747.21; 747.29 | Q25.4 |
|  | Congenital malformation of aorta unspecified |  | Q25.40 |
|  | Absence and aplasia of aorta |  | Q25.41 |
|  | Hypoplasia of aorta |  | Q25.42 |
|  | Congenital aneurysm of aorta |  | Q25.43 |
|  | Congenital dilation of aorta |  | Q25.44 |
|  | Double aortic arch |  | Q25.45 |
|  | Tortuous aortic arch |  | Q25.46 |
|  | Right aortic arch |  | Q25.47 |
|  | Anomalous origin of subclavian artery |  | Q25.48 |
|  | Other congenital malformations of aorta |  | Q25.49 |
|  | Atresia of pulmonary artery | 747.31 | Q25.5 |
|  | Stenosis of pulmonary artery | 747.39 | Q25.6 |
|  | Other congenital malformations of pulmonary artery |  | Q25.7 |
|  | Coarctation of pulmonary artery | 747.31 | Q25.71 |
|  | Congenital pulmonary arteriovenous malformation | 747.32 | Q25.72 |
|  | Other congenital malformations of pulmonary artery | 747.39 | Q25.79 |
|  | Other congenital malformations of other great arteries | 747.29 | Q25.8 |
|  | Congenital malformation of great arteries, unspecified | 747.29 | Q25.9 |
|  | Congenital malformations of great veins |  | Q26 |
|  | Congenital stenosis of vena cava | 747.49 | Q26.0 |
|  | Persistent left superior vena cava | 747.49 | Q26.1 |
|  | Total anomalous pulmonary venous connection | 747.41 | Q26.2 |
|  | Partial anomalous pulmonary venous connection | 747.42 | Q26.3 |
|  | Anomalous pulmonary venous connection, unspecified | 747.42 | Q26.4 |
|  | Anomalous portal venous connection | 747.61 | Q26.5 |
|  | Portal vein-hepatic artery fistula | 747.61 | Q26.6 |
|  | Other congenital malformations of great veins | 747.49 | Q26.8 |
|  | Congenital malformation of great vein, unspecified | 747.40 | Q26.9 |
|  | Other congenital malformations of peripheral vascular system | 747.5  747.62  747.62  747.89  747.63  747.64  747.61  747.62  747.69  747.89  747.69  747.60 | Q27.x |
|  | Other congenital malformations of circulatory system | 747.89  747.89  747.81  747.81  747.89  747.9 | Q28.x |
| **Neuromuscular disorders** | Anencephaly and similar malformations | 740.x | Q00.x |
|  | Encephalocele | 742.0 | Q01.x |
|  | Microcephaly | 742.1 | Q02.x |
|  | Congenital hydrocephalus | 742.3 | Q03.x |
|  | Other congenital malformations of brain | 742.2; 742.4; 742.9 | Q04.x |
|  | Spina bifida | 741.x | Q05.x |
|  | Other congenital malformations of spinal cord | 742.51; 742.59; 742.53; 742.9 | Q06.x |
|  | Other congenital malformations of nervous system | 741.90; 741.00; 742.8; 742.9 | Q07.x |
|  | Myalgia and myositis, unspecified | 729.1 | M60.9; M79.1; M79.7 |
|  | Diseases of the nervous system | 320.xx- 359.xx | G00.xx-G99.xx |
| **Bronchopulmonary dysplasia** | Bronchopulmonary dysplasia originating in the perinatal period | 770.7 | P27.1 |
| **Down syndrome** | Down syndrome |  | Q90 |
|  | Trisomy 21, nonmosaicism (meiotic nondisjunction) | 758.0 | Q90.0 |
|  | Trisomy 21, mosaicism (mitotic nondisjunction) | 758.0 | Q90.1 |
|  | Trisomy 21, translocation | 758.0 | Q90.2 |
|  | Down syndrome, unspecified | 758.0 | Q90.9 |
| **Immunodeficiency** | Immunodeficiency with predominantly antibody defects | 279.04;  279.00  279.01  279.03  279.02  279.05  279.19  279.09  279.19  279.19 | D80.x |
|  | Combined immunodeficiencies | 279.2  279.2  279.2  277.2  279.13  277.2  279.2  279.2  277.6  266.2  266.2  279.2  279.2 | D81.x |
|  | Immunodeficiency associated with other major defects | 279.12  279.11  279.8  279.8  279.8  279.8  279.8 | D82.x |
|  | Common variable immunodeficiency | 279.06  279.10  279.06  279.06  279.06 | D83.x |
|  | Other immunodeficiencies | 279.8  277.6  279.8  279.3  279.3 | D84.x |
|  | Other disorders involving the immune mechanism, not elsewhere classified | 273.0  273.2  273.1  289.89  279.8  279.51  279.52  279.53  279.50  279.41  279.8  279.9 | D89.x |
|  | Human immunodeficiency virus [HIV] disease | 042 | B20 |
| **Velo-cardio-facial syndrome** | Velo-cardio-facial syndrome | 758.32 | Q93.81 |
| **Congenital anomalies of respiratory system** | Congenital anomalies of respiratory system | 748.x | Q30.x – Q34.x |
| **Other congenital musculoskeletal anomalies** | Other congenital musculoskeletal anomalies | 756.x | Q75.x – Q79.x |
| **Cystic fibrosis with pulmonary manifestations** | Cystic fibrosis | 277.0 | E84 |
|  | Cystic fibrosis with pulmonary manifestations | 277.02 | E84.0 |
|  | Cystic fibrosis with intestinal manifestations |  | E84.1 |
|  | Meconium ileus in cystic fibrosis | 277.01 | E84.11 |
|  | Cystic fibrosis with other intestinal manifestations | 277.03 | E84.19 |
|  | Cystic fibrosis with other manifestations | 277.09 | E84.8 |
|  | Cystic fibrosis, unspecified | 277.00 | E84.9 |

**Table S2 Unit costs considered for each healthcare visit**

| **Healthcare visit** | **Unit cost** |
| --- | --- |
| Visit to a primary care doctor | €63.8^(a)^ |
| Visit to a primary care nurse | €34.4^(b)^ |
| Outpatient first visit | €223.4^(c)^ |
| Outpatient successive visit | €123.8^(d)^ |
| Emergency visit | €174.0^(e)^ |

1. eSalud original reference: Consejería de Sanidad y Políticas Sociales (2020). Resolución de 6 de febrero de 2020. Diario Oficial de Extremadura, número 28, 11 de febrero de 2020.[22]
2. Mean cost computed, using the cost for visit in regular working period of the center and outside of that period. eSalud original reference: Consejería de Salud (2018). Orden de 8 de mayo de 2018. Boletín Oficial de la Junta de Andalucía núm. 92, 15 de mayo de 2018.[22] Original costs were updated to 2020 costs using the yearly change in the consumer price index published by the National Statistics Institute of Spain.[35]
3. Mean cost computed, using the cost for first outpatient visit (*consulta externa*) from two eSalud original references: 1. Consejería de Sanidad y Políticas Sociales (2020). Resolución de 6 de febrero de 2020. Diario Oficial de Extremadura, número 28, 11 de febrero de 2020; 2. Osakidetza-Servicio Vasco de Salud (2020). Acuerdo del Consejo de Administración de 19 de diciembre de 2019. Boletín Oficial del País Vasco, nº 21, 31 de enero de 2020.[22]
4. Mean cost computed, using the cost for subsequent outpatient visits (*consulta externa*) from two eSalud original references: 1. Consejería de Sanidad y Políticas Sociales (2020). Resolución de 6 de febrero de 2020. Diario Oficial de Extremadura, número 28, 11 de febrero de 2020; 2. Osakidetza-Servicio Vasco de Salud (2020). Acuerdo del Consejo de Administración de 19 de diciembre de 2019. Boletín Oficial del País Vasco, nº 21, 31 de enero de 2020.[22]
5. eSalud original reference: Osakidetza-Servicio Vasco de Salud (2020). Acuerdo del Consejo de Administración de 19 de diciembre de 2019. Boletín Oficial del País Vasco, nº 21, 31 de enero de 2020.[22]
